# Associations between IL-6 and Echo-Parameters in Patients with Early Onset Coronary Artery Disease

**DOI:** 10.3390/diagnostics9040189

**Published:** 2019-11-14

**Authors:** Natalia Pauli, Kamila Puchałowicz, Agnieszka Kuligowska, Andrzej Krzystolik, Violetta Dziedziejko, Krzysztof Safranow, Michał Rać, Dariusz Chlubek, Monika Ewa Rać

**Affiliations:** 1 Department of Cardiology, Regional Hospital, 66-400 Gorzow Wielkopolski, Poland; laurapalmer81@wp.pl; 2 Department of Biochemistry and Medical Chemistry, Pomeranian Medical University, 70-111 Szczecin, Poland; kamila.puchalowicz@pum.edu.pl (K.P.); agnieszka.kaleta91@gmail.com (A.K.); viola@pum.edu.pl (V.D.); chrissaf@mp.pl (K.S.); dchlubek@pum.edu.pl (D.C.); 3 Department of Cardiology, County Hospital, 71-455 Szczecin, Poland; akrzystolik@poczta.onet.pl; 4 Department of Diagnostic Imaging and Interventional Radiology, Pomeranian Medical University, 71-252 Szczecin, Poland; hno15hno@gmail.com

**Keywords:** atherosclerosis, cardiovascular disease, echocardiography, ejection fraction, shortening fraction, inflammation, interleukin, myocardial infarction, systolic function

## Abstract

Background: Over the last two decades, many studies have investigated the association between interleukin 6 (IL-6) and pathogenesis and progression of coronary artery disease (CAD). Patients with CAD manifested at a young age are a particularly interesting group. They differ from older patients, not only in terms of the severity of coronary artery atherosclerosis, but also risk factor profiles, short- and long-term prognosis after myocardial infarction (MI). The role of IL-6 in younger patients with CAD is less well-known. Therefore, our study aimed to analyze the relationship between IL-6 level and other inflammations, atherosclerosis, and cardiac function parameters in early onset CAD patients. Methods: The study covered 100 patients with early onset CAD and a group of 50 healthy participants. Plasma levels of IL-6 and basic biochemical parameters, anthropometric, echocardiographic, and arteries Doppler ultrasound measurements were performed. Results: We did not observe a significant difference in IL-6 concentration in plasma between patients with early onset CAD and a control group, but IL-6 level was negatively correlated with echocardiographic measurements of ascending aorta diameter, left ventricular shortening fraction, and right ventricular end-diastolic diameter in our patients. Conclusions: In patients with early onset CAD, plasma IL-6 level is associated with other inflammation parameters and with cardiac function, potentially contributing to right ventricular remodeling and left ventricular systolic dysfunction. This suggests possible prognostic benefits of long-time observation of IL-6 level after the acute coronary syndrome.

## 1. Introduction

Coronary artery disease (CAD) has become a major public health challenge. It is related to high morbidity and mortality worldwide and it is increasingly prevalent in the Western world [1]. Annually, about 170,000 people die from cardiovascular disease in Poland, which is 47% of all deaths [2]. Although this disorder usually affects the elderly, an increasing prevalence of CAD among younger people has been observed over the last few decades. The manifestation of CAD at a young age is defined as premature or early onset CAD, but various studies recognize the upper age limit in the range of 35 to 55 years [3]. In young patients with acute coronary events, revascularization is associated with low in-hospital and short-term mortality, as well as low mortalities in follow-up of six months up to seven years. However, mortality in young CAD group increases after a longer time period. Individuals with premature CAD have overall poor prognosis: patients died within 15 years of initial data collection [4]. A past myocardial infarction (MI), diabetes, smoking, and lower ejection fraction predict a significantly higher mortality. Better knowledge of this group of patients is important in the era of preventive cardiology. Understanding the significance of markers of oxidative stress, endothelial dysfunction, and inflammation in pathophysiology of early onset CAD may help define new aspects of vascular biology and additional markers of future risk [4]. Primary and secondary prevention play an important role in avoidance of initial and further coronary events [3].

The pathogenesis of premature CAD involves many conventional (hypertension, diabetes, dyslipidemia, obesity, smoking) and newer risk factors [4]. Inflammation is increasingly seen as a new modifiable cardiovascular risk factor [5]. Many studies have shown that inflammation plays a role in the pathophysiology of atherosclerosis and CAD [6,7,8]. Molecular mechanisms underlying inflammation-associated diseases include changes of immune response and normal cell microenvironment resulting from an imbalance of inflammatory cytokines [6]. Inflammatory markers are important mediators in the multi-step cascade of atherosclerosis, which finally leads to the rupture of the atherosclerotic plaque [9].

Interleukin 6 (IL-6) is an important proatherogenic cytokine [10]. This multifunctional proinflammatory cytokine is produced by T lymphocytes, macrophages, and adipocytes, and acts via its membrane-bound (IL-6R) or soluble receptors (sIL-6R) [5]. The key mechanisms by which IL-6 contributes to the development of CAD were summarized by Yudkin et al. [11]. It is a central mediator of acute phase response by stimulation of the liver to produce acute phase proteins, such as C-reactive protein (CRP), plasma amyloid A (SAA), and fibrinogen. The acute phase response is associated with increased blood viscosity and increased number and activity of platelets. Furthermore, HDL-cholesterol level is reduced by raised SAA. Autocrine and paracrine activation of monocytes by IL-6 in the vessel wall contributes to the deposition of fibrinogen. IL-6 also decreases lipoprotein lipase activity and monomeric lipoprotein lipase levels in plasma, which increases macrophages uptake of lipids that results in foam cell formation. Circulating IL-6 affects the endocrine system—it stimulates the hypothalamic–pituitary–adrenal axis, the activation of which is associated with central obesity, hypertension, and insulin resistance [11]. Accordingly, IL-6 is an important mediator of atherosclerotic disease and is associated with CAD and acute ischemic conditions [12,13].

However, not only does IL-6 take part in the pathogenesis of CAD, but also its levels can be affected by CAD and its consequences. This cytokine is a promising noninvasive marker for monitoring patients with CAD before and after acute coronary syndrome. Recent studies have found associations between IL-6 level and CAD severity [14,15], all-cause and cardiovascular mortality in patients with CAD [12], long-term cardiovascular mortality in patients with ST-segment elevation MI [16], and progression to heart failure [17]. However, the role of IL-6 in younger patients with CAD is less well-known. Therefore, the present study aimed to analyze the relationship between IL-6 level and other inflammations, atherosclerosis, and cardiac function parameters in early onset CAD patients.

## 2. Materials and Methods

### 2.1. Study and Control Group

The study group covered 100 clinically stable patients with early onset CAD: 75 men aged up to 50 years and 25 women aged up to 55 years at the moment of CAD diagnosis. All patients were Polish residents treated in the Department of Cardiology of the County Hospital in Szczecin (northwestern Poland). They did not have acute coronary syndrome or revascularization therapy within the last month and received optimal pharmacological treatment. The diagnosis of CAD was based on the following criteria: Angiographically documented presence of at least one coronary lesion (≥40% diameter stenosis of the left main coronary artery or ≥50% stenosis of one of the three major epicardial arteries, or ≥70% stenosis of a branch), a history of revascularization therapy, or past MI. The study excluded patients with hemodynamically significant congenital or acquired valvular heart disease, symptomatic heart failure (NYHA class > I), severe renal failure (creatinine >3 mg/dL), thyroid dysfunction (current hypo- or hyperthyroidism), type 1 diabetes mellitus, or malignancy. The biochemical control group included 50 healthy subjects (without CAD) with the same exclusion criteria as in the study group and equipotency to the age and sex of study group (74% males; average 48 ± 3.20 years). It was a randomly selected control group from among persons reporting for periodic medical check-ups at the Occupational Medicine Clinic. All subjects gave their informed consent for inclusion before they participated in the study. The study was conducted in accordance with the Declaration of Helsinki, and was approved by the Pomeranian Medical University Ethics Committee (BN-001/162/04, approval date: 6 November 2017).

### 2.2. Blood Samples and Biochemical Measurements

The fasting blood samples were collected from the study and control subjects for complete blood count, plasma glucose, lipid profile (total, high- and low-density lipoprotein cholesterol, and triglycerides), apolipoproteins (ApoA1, ApoB, Lp_a_)m and high-sensitivity C-reactive protein (CRP) measurements. The tests were carried out on a Roche Cobas 6000 analyzer.

### 2.3. Measurement of IL-6 Concentration

The plasma concentrations of human IL-6 were determined using a commercially available ELISA kit (Quantikinine R&D Systems Inc., UK) according to the manufacturer’s protocol. Absorbance was read at 450 nm (with correction at λ = 540 nm) using automated Microplate Reader EnVision 2104 (Perkin Elmer). Plasma samples were stored at −80 °C until analyzed.

### 2.4. Anthropometric Parameters

The following clinical parameters were measured for each patient: weight, height, waist and hip circumference, systolic and diastolic blood pressure. The measurements were followed by the calculations of the body mass index (BMI), waist-to-hip ratio (WHR), and mean arterial pressure (MAP).

### 2.5. Echocardiography

Echocardiographic assessment of cardiac anatomy and function was performed in each patient on an ultrasound unit (Medison SA 9900). End-diastolic and end-systolic volumes assessed using the biplane Simpson’s method were used to calculate left ventricular ejection fraction (LVEF). Left ventricular mass (LVM) was calculated using the Devereux equation [18]. Left ventricular mass index (LVMI) was calculated by dividing LVM by body surface area [19]. To evaluate left ventricle diastolic function, we used mitral flow pulse wave Doppler imaging with evaluation of E/A ratio and Tissue Doppler Imaging (TDI) with early diastolic mitral annular velocity measurement and evaluation of septal E’, A’, and E’/A’ ratio. As a criterion for normal diastolic function, we used values of E/A 1–2.5 and E’/A’ >1, impaired diastolic function: E/A <1 and E’/A’ <1, pseudonormal diastolic function: E/A 1–2.5 and E’/A’ <1, restriction: E/A >2.5 and E’/A’ >1. None of the patients met the criteria for restriction.

### 2.6. Doppler Ultrasound of Carotid and Peripheral Arteries

Doppler ultrasound of carotid and peripheral arteries was also carried out in each patient. Intima-media complex thickness (IMC) of common carotid (CCA) and brachial arteries, density and thickness of atheromatous plaque at CCA bifurcation were measured with the Math program on the Doppler ultrasound unit (Technos, Esaote). Density measurement was based on the intensity of reflection from the plaque, dependent on calcium deposits. Ankle-brachial index (ABI) was calculated by dividing the systolic blood pressure in the arteries at the left and right ankle (posterior tibial artery) or foot (anterior tibial artery) by the higher of the two systolic blood pressures in the arms.

### 2.7. Statistical Analysis

The values are presented as frequency (percentage) or mean ± standard deviation (SD). The comparison of biochemical parameters of the study and control groups and associations between qualitative variables and IL-6 level were done using the Mann–Whitney *U* test, whilst correlations between plasma IL-6 concentration and quantitative variables were assessed with the Spearman rank correlation coefficient (Rs). Multivariate analysis performed with general linear model (GLM) included age, sex, BMI, history of arterial hypertension, past MI, and logarithm of plasma IL-6 concentration as independent variables, and one of echocardiographic parameters as dependent variable. Standardized β was presented as a measure of association with individual independent variables and *R*^2^ was calculated as a measure of fit of the whole GLM. *p*-values <0.05 were considered statistically significant. Bonferroni correction for multiple comparisons was used where indicated.

## 3. Results

### 3.1. The Characteristics of the Group of Patients with Early Onset CAD

The clinical characteristics of the study group are shown in Table 1.

### 3.2. The Comparison of Biochemical Parameters of the Study and Control Groups

Patients with early onset CAD had significantly higher complete blood count parameters—white blood cells (WBC), red blood cells (RBC), hemoglobin, hematocrit, mean corpuscular hemoglobin concentration (MCHC), red cell distribution width (PDW), mean platelet volume (MPV), and platelet large cell ratio (PLCR)—than the control participants. Plasma high-sensitivity C-reactive protein (hsCRP), total, high- and low-density lipoprotein cholesterol, ApoA1, and ApoB concentrations were significantly lower, and triglycerides significantly higher in the study group than the control group. The hsCRP parameter was higher in the control group than CAD, probably because almost all of the CAD group was statin-treated. No statistically significant difference in plasma IL-6 concentrations between the study and control groups was observed. The comparison of biochemical parameters of the study and the control group is presented in Table 2. In the control group, IL-6 level positively correlated with hsCRP (Rs = 0.70, *p* = 0.042), hemoglobin (Rs = 0.30, *p* = 0.032), hematocrit (Rs = 0.31, *p* = 0.025), and monocytes (Rs = 0.34, *p* = 0.015).

### 3.3. The Correlations between Plasma IL-6 Concentration and Biochemical and Clinical Parameters in Patients with Early Onset CAD

The Spearman rank correlation coefficient (Rs) in the early onset CAD patients showed statistically significant correlations between IL-6 plasma concentration and quantitative variables. A history of MI is a factor that affects many echocardiographic parameters. Therefore, in order to verify the possible impact of the past infarction on the obtained test results, an additional analysis was performed with the division of CAD patients into subgroups of people with and without infarction. Male sex is also a known factor predisposing to coronary artery disease, which was reflected in a high percentage of men among the studied patients. Therefore, in order to verify the possible impact of the patient’s gender on the obtained test results, an additional analysis was performed with the division of CAD patients into male and female subgroups. In the CAD group, there were no significant differences between plasma IL-6 levels in the male and female groups, also in the post-MI and without MI groups. The analysis with Bonferroni correction for multiple comparisons revealed positive correlations between IL-6 and WBC and hsCRP as statistically significant. Significantly negative correlations (*p* < 0.05) were demonstrated between IL-6 and MCHC, echocardiographic measurements of ascending aorta diameter, left ventricular shortening fraction, right ventricular end-diastolic diameter, and IMC left brachial artery performed in the whole study group and in the subgroup of males and the subgroup post-MI. Rs values only for parameters with statistically significant correlations are presented in Table 3.

**Figure 1:**
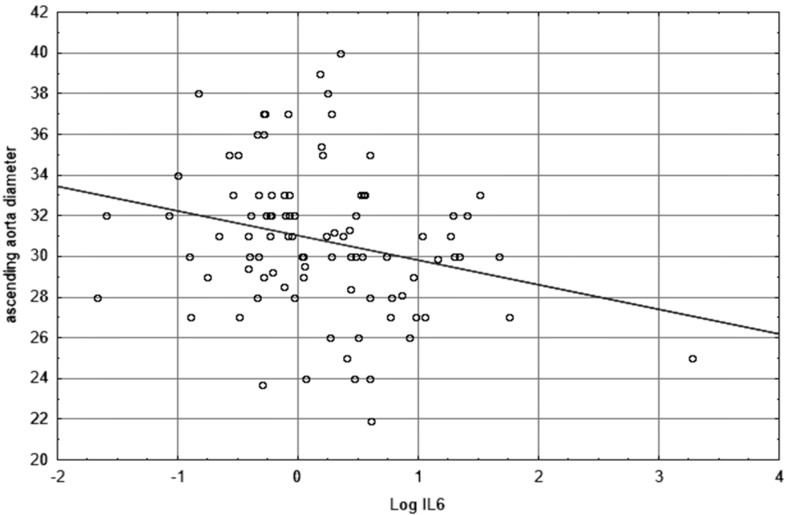
The scatterplot showing the relationship between Log IL-6 and ascending aorta diameter.

**Figure 2:**
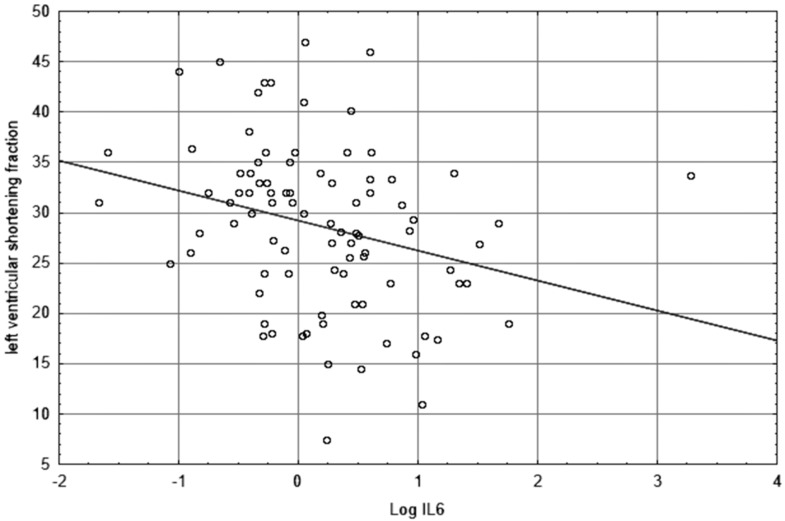
The scatterplot showing the relationship between Log IL-6 and left ventricular shortening fraction.

The graphic representations of the relationship between log IL-6 and ascending aorta diameter or left ventricular shortening fraction are shown in Figure 1 and Figure 2, respectively.

The significant independent predictors of higher aorta diameter were: lower IL-6 level (β = −0.22, *p* = 0.016), male gender (β = 0.44, *p* = 0.000011), older age (β = 0.25, *p* = 0.007), and higher BMI (β = 0.23, *p* = 0.022), with R^2^ = 0.37 for the whole multivariate model. The significant independent predictors associated with lower left ventricular shortening fraction were higher IL-6 level (β = −0.27, *p* = 0.008) and past MI (β = −0.33, *p* = 0.002) with R^2^ = 0.21. The only significant independent predictor of higher right ventricular end-diastolic diameter was younger age (β = −0.24, *p* = 0.03), while IL-6 plasma concentration showed no significant association (β = −0.09, *p* = 0.38). Other clinical parameters of atherosclerosis or cardiac function in patients with early onset CAD did not correlate with IL-6 level (Table S1). Moreover, significant associations between qualitative variables and plasma IL-6 concentration in early onset CAD patients were not found (Table S2).

## 4. Discussion

Involvement of inflammation parameters in the pathogenesis of CAD has been intensively studied in recent years. Our results pointed out that the role of IL-6 in younger patients with CAD is inconclusive. A rapid decrease of IL-6 levels occurs a few weeks after MI. This cytokine may be a noninvasive marker for monitoring patients with CAD during the acute coronary syndrome, as other studies have found. However, we found that one month or more after the cardiovascular incident, plasma IL-6 levels do not differ between CAD and healthy people, although still might contribute to right ventricular (RV) remodeling or left ventricular (LV) systolic dysfunction. The mechanism of this phenomenon is unclear. We did not observe a statistically significant difference in plasma IL-6 concentration between patients with early onset CAD and control participants, but IL-6 level was higher in the CAD group. Based on the available literature data, there are no precise reference ranges for IL-6. Todd et al. [20] determined that the mean values for the plasma in healthy subjects was 1.89 pg/mL and upper limits of the reference interval were 4.45 pg/mL (95th percentile) and 7.72 pg/mL (99th percentile). Comparable results were obtained by Hennø et al. [21]. This indicates that the IL-6 level measured in our early onset CAD patients was normal. There are particular factors affecting the IL-6 level in CAD patients, such as co-occurrence of risk factors and condition of patients, age, smoking, diabetes, hypertension, dyslipidemia, body mass index, and inflammation [22]. It should be noted that IL-6 has a predictive value for diagnosing the presence of early CAD and it improves the accuracy of traditional risk scores. Its level above 1 pg/mL is highly predictive for CAD in patients at intermediate atherosclerotic cardiovascular risk with chest pain [1]. It is possible that young age of patients, low percentage of smokers, normalized lipid profile, fasting glucose level, and blood pressure are some of the causes for IL-6 concentrations in our CAD group. It was shown that IL-6 is an indicator of the severity of atherosclerotic disease [1,9,14,15,23] and myocardial damage during acute coronary syndrome [24]. Mowafy et al. [9] emphasized that IL-6 level positively correlates with severity of the clinical condition and number of affected vessels in patients with acute coronary syndrome. It is proportional with severity of the lesions in the coronary arteries. IL-6 level in patients with significant angiographically lesions was significantly higher than in patients with non-significant lesions or the control group (45.5 pg/mL vs. 9.22 pg/mL vs. 3.83 pg/mL, respectively; *p* < 0.001) [9]. Several studies indicate that the highest IL-6 levels are reached on the first day after MI (as a result of myocardial necrosis), and thereafter, a rapid decline occurs, obtaining a stable level after a few weeks (depending on the source from 2 weeks to 6 months) [24,25,26]. Furthermore, it does not change significantly over time [24,26,27]. Among survivors of MI, IL-6 levels are associated with traditional cardiovascular risk factors, such as age, pack-years of smoking, HDL cholesterol, or systolic blood pressure [27]. That is why we suspect that a similar IL-6 level in both of our groups may be also due to a good condition of patients (they did not have acute coronary syndrome or revascularization procedures within a month before the study) and received effective treatment including drug therapy and lifestyle changes after CAD diagnosis. Presumably, one of the causes may have been the statin therapy, as 96% of our patients received statins. There are reports of reductions in circulating IL-6 concentrations in patients taking statins [28,29]. They decrease the production and release of IL-6 in the endothelium and leukocytes [28,30]. Different types of statins vary in their potency of IL-6 release inhibition and the type of cells they affect [31,32]. Statins, apart from LDL-cholesterol lowering, exert pleiotropic effects on endothelial function, vascular inflammation, immunomodulation, and thrombogenesis, and therefore they prevent cardiovascular events, ameliorate the prognosis of patients affected by acute MI, as well as reduce the risk of restenosis after angioplasty [29]. Statins may have an anti-inflammatory effect on hsCRP level [33]. Another group of drugs, angiotensin-converting enzyme inhibitors (ACEI), may have also contributed to the reduction of the IL-6 level (80% of our patients received ACEI) [34,35]. Furthermore, regular physical exercise is thought to reduce IL-6 level and ischemic events in men with stable coronary artery disease [36]. Involvement of inflammation in the pathogenesis of CAD and its complications has been intensively studied over the last 20 years. Therefore, inflammation parameters, for example IL-6 or hsCRP, have become potential markers for monitoring condition of patients with CAD. Unsurprisingly, we observed that IL-6 level positively correlates with other inflammatory parameters hsCRP and WBC, which is consistent with the results of other studies [19]. The levels of IL-6 [1,15,37] and hsCRP [38,39] could help predict the angiographic severity of CAD. It is well-known that these inflammatory markers have been linked to coronary events. Patients with high circulating levels have a higher risk of cardiovascular events [1,15].

We evaluated correlations between IL-6 and radiological parameters of atherosclerosis. IL-6 is an early and central regulator of inflammation found in high abundance in atherosclerotic plaques. Its atherogenic action results from an induction of acute-phase proteins, stimulation of vascular smooth muscle proliferation, activation of platelets, chemotactic activity for macrophages and neutrophils, ability to activate endothelial cells and induce chemokine production and adhesion molecule expression. In this way, it participates in lipid processing and plaque formation [10]. However, we did not find any positive correlations between IL-6 level and severity of atherosclerosis measured as IMC thickness of common carotid and brachial arteries or density and thickness of atheromatous plaque at CCA bifurcation. The population-based study conducted by Eltoft et al. [40] demonstrated IL-6 as a predictor of progressive atherosclerotic disease. The IL-6 level positively correlated with the presence of unstable plaque, dynamics of changes in atherosclerotic plaque morphology in the internal carotid artery [41], and the degree of carotid stenosis [41,42]. Nonetheless, current studies differ as to whether there is an association between IL-6 and carotid IMC thickness, which is the parameter used to detect and monitor early atherosclerotic lesions [43]. The associations between IL-6 level and IMC thickness of carotid bulb and internal carotid artery [42], and carotid IMC thickness [44,45] were demonstrated by several groups of researchers. However, these results were not confirmed in other studies after adjustment for conventional risk factors [46,47]. What is important, Okazaki et al. [48] found a significant association between the long-term mean concentration of IL-6 (measures repeated three times every 3 years), but no baseline concentration, and the increase of carotid IMC thickness. The authors stressed the importance of long-term mean concentration of IL-6 in studies on atherosclerotic lesions (early, not advanced). IL-6 is a short-acting cytokine susceptible to change, thus it is difficult to establish its association with progression of atherosclerotic lesions based on a single measurement. Furthermore, positive correlations between IL-6 and markers of atherosclerosis were mainly observed in patients older than ours. Carotid IMC thickness positively correlates with advancing atherosclerotic lesions in coronary arteries [49] that are usually less extensive in young patients than older ones [50]. However, we demonstrated the negative correlation of IL-6 level with diameter of ascending aorta. Such results were clarified by Li et al. [51] in echocardiographic examination on mice, which showed a thickened vascular wall and narrowed aorta diameter in correlation with increased cytokines in vessels. The authors demonstrated dysfunction of endothelial cells, progressive fibrosis in the vessel, which might have been associated with mitochondrial dysfunction in response to higher IL-18 level, which is a proinflammatory cytokine. In that case, ATP productivity and activity of respiratory electron transport chain complex were decreased in the mitochondria of aorta, and production of reactive oxygen species was augmented.

It is important to take into account that young post-MI patients are different from older patients, not only in terms of the severity of coronary artery atherosclerosis, but also risk factor profiles (higher prevalence of smoking, hyperlipidaemia, family history of premature CAD, and male gender) and short- (better) and long-term (unfavorable) prognosis [27,52]. The changes in the cardiovascular system in young post-MI patients may be related to IL-6 level. Echocardiographic evaluation of left ventricular (LV) function has significant implications for the prognosis of heart failure and mortality in patients after MI. We showed a negative correlation between IL-6 and echocardiographic parameters of LV systolic function, shortening fraction (SF). Also, LVEF inversely correlated with IL-6, but it was not significant (*p* = 0.095). A strong inverse relationship between IL-6 level and LV systolic function was confirmed in individuals with [17] and without clinical cardiovascular disease [53]. Other authors [17,54] indicate that plasma IL-6 level negatively correlates with LVEF and positively with LV end-diastolic diameter in patients with stable CAD. They found an association between IL-6 concentrations and the extent of asymptomatic LV systolic dysfunction, and its diagnostic value in predicting progression to heart failure during follow-up. Elevated IL-6 levels were independently associated with LV hypertrophy. Chronic IL-6 signaling activation in heart underlies the pathogenetic link between inflammation and LV dysfunction, which leads to reduced contractility and contributes to the progression of compensatory LV hypertrophy to heart failure [52,55].

Inflammatory mediators are also upregulated in the right ventricle in response to pressure overload, along with accumulation of inflammatory cells. However, the exact role of the inflammatory mediators in this context has yet to be established [56]. We found a negative correlation between IL-6 and right ventricular end-diastolic diameter. Recently, relationships between increased circulating IL-6 levels and right ventricular (RV) function in pulmonary arterial hypertension patients have been analyzed [57]. In the cited study, plasma IL-6 levels inversely correlated with echocardiography derived measures of RV function, including RV fractional area change. Interestingly, in a recent study, a strong, independent, inverse relationship between IL-6 and RV morphology has been demonstrated also in asymptomatic individuals without documented cardiovascular disease [58]. Further prospective studies on a larger group of patients with CAD are required to gain mechanistic insight into how these mediators contribute to maladaptive RV remodeling.

Our study has some limitations. The measurements were taken only once. Danesh et al. [22] emphasize that within-person variability in IL-6 levels is high and serious underestimation of the associations is highly likely. Paired IL-6 measurements enabled approximate correction for within-person variability. The long-term average IL-6 value can be a better quantitative marker of chronic inflammatory conditions than a single measurement of the baseline IL-6 level. Moreover, the number of cases in both groups was relatively small. There are also other promising predictors of cardiovascular events, which were not assessed in this study: Arterial stiffness and early arterial aging, known to be associated with interleukins and inflammatory markers [59]. Moreover, a healthy diet, especially the inclusion of sufficient fruits and vegetables in the diet, is regarded as significantly important and may exert several cardioprotective effects [60].

## 5. Conclusions

In conclusion, in patients with early onset CAD, plasma IL-6 level is associated with other inflammation parameters and with cardiac function, potentially contributing to right ventricular remodeling and left ventricular systolic dysfunction. This suggests possible prognostic benefits of long-time observation of IL-6 level after the acute coronary syndrome.

## Figures and Tables

**Table 1:** The characteristics of the study group (*n* = 100).

Parameter	Value
Gender (% males)	75%
Age of patients (years)	49.9 ± 5.91
Waist (cm)	98.3 ± 12.5
Hip (cm)	103 ± 9
WHR	0.96 ± 0.09
BMI (kg/m^2^)	28.1 ± 4.0
Systolic BP (mmHg)	127 ± 14.0
Diastolic BP (mmHg)	77.0 ± 9.0
MAP (mmHg)	93.8 ± 9.4
History of hypertension	66%
Age at diagnosis of hypertension (years)	42.6 ± 8.6
Past MI	70%
Age of the first MI (years)	44.0 ± 5.6
Time since diagnosis of MI to joining the program (years)	3.20 ± 0.74
Current smoking	15%
Past smoking	89%
Years smoking	18.9 ± 9.8
Past PTCA	71%
Past CABG	37%
ACEI	80%
ARB	17%
Beta-blockers	88%
Diuretics	31%
Calcium channel blockers	18%
Statins	96%

ACEI—angiotensin 1 converting enzyme inhibitors, ARB—angiotensin 2 receptor blockers, BMI—body mass index, BP—blood pressure, CABG—coronary artery bypass grafting, MAP—mean arterial pressure, MI—myocardial infarction, PTCA—percutaneous transluminal coronary angioplasty, WHR—waist-to-hip ratio.

**Table 2:** The biochemical parameters of study and control group.

Parameter	Study Group (Mean ± SD)	Control Group (Mean ± SD)	*p*-Value
IL-6 (pg/mL)	1.69 ± 2.77	1.47 ± 0.33	0.194419
WBC (G/L)	6.80 ± 0.22	5.50 ± 0.16	**0.000660**
RBC (T/L)	4.96 ± 0.04	4.64 ± 0.06	0.000205 *
Hemoglobin (g/dL)	15.0 ± 0.12	13.7 ± 0.15	0.000001 *
Hematocrit (%)	43.9 ± 0.33	41.4 ± 0.43	0.000011 *
MCV (fL)	89.5 ± 0.45	90.0 ± 0.72	0.964125
MCH (pg/erythrocyte)	30.1 ± 0.18	30.0 ± 0.27	0.314198
MCHC (g/dL)	33.7 ± 0.09	33.3 ± 0.13	**0.008262**
Platelets (G/L)	216 ± 4.58	223 ± 11.2	0.599570
Neutrophils (%WBC)	54.5 ± 0.92	54.4 ± 1.48	0.476875
Lymphocytes (%WBC)	33.3 ± 0.82	34.1 ± 1.43	0.616375
Monocytes (%WBC)	9.10 ± 0.20	8.50 ± 0.27	0.078655
Eosinophils (%WBC)	2.10 ± 0.19	2.90 ± 0.43	**0.021493**
Basophils (%WBC)	0.30 ± 0.02	0.50 ± 0.04	**0.001138**
RDW	13.3 ± 0.11	13.5 ± 0.13	0.205517
PDW	13.1 ± 0.23	12.4 ± 0.31	**0.001486**
MPV	10.6 ± 0.09	10.3 ± 0.14	**0.006729**
PCT	0.23 ± 0.01	0.22 ± 0.01	0.616375
PLCR	30.5 ± 0.62	27.6 ± 1.00	**0.003356**
hsCRP (mg/L)	1.20 ± 0.27	2.10 ± 0.26	**0.002557**
Glucose (mg/dL)	101 ± 2.49	96.0 ± 4.07	0.121613
Total cholesterol (mg/dL)	163 ± 4.06	217 ± 6.36	0.000002 *
HDL (mg/dL)	47.0 ± 1.16	66.0 ± 2.15	0.000001 *
LDL (mg/dL)	93.0 ± 3.64	126 ± 5.74	**0.000680**
TG (mg/dL)	128 ± 5.74	110 ± 8.52	0.051383
LPa (mg/dL)	20.3 ± 4.96	23.2 ± 9.83	0.532188
ApoA1 (mg/dL)	146 ± 3.85	177 ± 5.16	0.000012 *
ApoB (mg/dL)	74.0 ± 2.25	92.0 ± 3.78	**0.003908**
ApoB/ApoA1	0.52 ± 0.02	0.51 ± 0.03	0.965446

ApoA1, ApoB, and Lp(a)—apolipoproteins, HDL—high-density lipoprotein cholesterol, hsCRP—high-sensitivity C-reactive protein, IL-6—interleukin 6, LDL—low-density lipoprotein cholesterol, MCH—mean corpuscular hemoglobin, MCHC—mean corpuscular hemoglobin concentration, MCV—mean corpuscular volume, MPV—mean platelet volume, PCT—plateletcrit, PDW—platelet distribution width, PLCR—platelet large cell ratio, RBC—red blood cells, RDW—red cell distribution width, SD—standard deviation, TG—triglycerides, WBC—white blood cells. Significant associations (*p* < 0.05) are marked in bold. * Statistically significant with Bonferroni correction for multiple comparisons.

**Table 3:** The correlations between plasma IL-6 concentration and quantitative parameters in early onset CAD patients in the whole study group and in the subgroups of males or females and in patients post- or without MI (only parameters with statistically significant or borderline correlations).

Parameter	Correlations for CAD Patients (*n* = 100)		Correlations for Males (*n* = 75)		Correlations for Females (*n* = 25)		Correlations for Patients Post-MI (*n* = 70)		Correlations for Patients without MI (*n* = 30)	
	Rs	*p*-value	Rs	*p*-value	Rs	*p*-value	Rs	*p*-value	Rs	*p*-value
Time since the onset of MI	0.09	0.48	−0.08	0.58	0.61	**0.021**	0.09	0.48	-	-
WBC	0.46	0.000003 *	0.53	0.000003 *	0.27	0.19	0.46	**0.0001**	0.46	**0.009**
MCHC	−0.32	**0.001**	−0.33	**0.005**	−0.12	0.58	−0.27	**0.031**	−0.47	**0.007**
hsCRP	0.52	0.000001 *	0.55	0.000001 *	0.40	0.062	0.47	**0.0002**	0.62	**0.0002**
Ascending aorta diameter	−0.26	**0.011**	−0.26	**0.032**	−0.04	0.84	−0.26	**0.043**	−0.31	0.09
RVEDD	−0.21	**0.041**	−0.12	0.33	−0.29	0.17	−0.30	**0.019**	−0.04	0.84
LVEF	−0.19	0.07	−0.22	0.07	−0.10	0.64	−0.13	0.30	−0.38	**0.034**
LVSF	−0.34	**0.001**	−0.33	**0.006**	−0.30	0.16	−0.37	**0.004**	−0.37	**0.034**
IMC lba mean	−0.30	**0.015**	−0.13	0.39	−0.27	0.27	−0.28	0.06	−0.37	0.09

hsCRP—high-sensitivity C-reactive protein, IMC lba—intima-media complex of left brachial artery—value calculated as mean of measurements of the left artery, LVEF—left ventricular ejection fraction, LVSF—left ventricular shortening fraction, MCHC—mean corpuscular hemoglobin concentration, MI—myocardial infarction, RVEDD—right ventricular end-diastolic diameter, WBC—white blood cells. Significant associations (*p* < 0.05) are marked in bold. * Statistically significant with Bonferroni correction for multiple comparisons.

## Supplementary Materials

The following are available online at https://www.mdpi.com/2075-4418/9/4/189/s1: Table S1. Spearman’s rank correlation coefficient (Rs) and probability (*p*) value for plasma IL-6 concentration and biochemical and clinical parameters in early onset CAD patients; Table S2. The associations between qualitative variables and plasma IL-6 concentration in early onset CAD patients.
