# Supplementary material for: Associations between IL-6 and Echo-Parameters in Patients with Early Onset Coronary Artery Disease
Source: Diagnostics (Basel). 2019 Nov 14;9(4):189. doi: 10.3390/diagnostics9040189 (PMC6963263; doi:10.3390/diagnostics9040189)
Supplement: Supplementary file 1 [file diagnostics-09-00189-s001.pdf]

Table S1. Sperman's rank correlation coefficient (Rs) and probability (p) value for serum IL-6 concentration and biochemical and clinical parameters in early onset CAD patients.

Parameter	IL-6	
	Rs	p-value
Age of patients	-0.011220	0.915460
Waist	0.010406	0.923789
Hip	-0.038176	0.725544
WHR	0.011717	0.914225
Body weight	-0.020530	0.846838
BMI	0.088573	0.403775
Systolic BP	-0.010310	0.923170
Diastolic BP	0.101771	0.339845
MAP	0.068154	0.523296
HR	-0.084947	0.450846
Age of the first MI	-0.017427	0.893059
**Times since the onset of MI**	**0.252287**	**0.044307**
**WBC**	**0.492151**	**0.000001**
RBC	-0.117451	0.264846
Hemoglobin	-0.100866	0.338723
Hematocrit	0.003569	0.973067
MCV	0.160129	0.127318
MCH	-0.007994	0.939713
**MCHC**	**-0.295951**	**0.004179**
RDW	0.160498	0.128570
%Neutrophils	0.138923	0.186602
%Lymphocytes	-0.187338	0.073746
%Monocytes	-0.000613	0.995373
%Eosinophils	-0.045754	0.664955
%Basophils	-0.094071	0.372423
Platelets	0.182934	0.080913
PDW	-0.053797	0.610528
MPV	-0.084068	0.425600
PCT	0.155168	0.139694
PLCR	-0.046963	0.658455
**hsCRP**	**0.526116**	**0.000000**
Glucose	-0.114907	0.270107
Total cholesterol	0.129366	0.213978
LDL	0.077411	0.458333
HDL	-0.041502	0.691248
Triacylglicerydes	0.186045	0.072598
LPa	-0.097841	0.348158
ApoB	0.076316	0.464733
ApoA1	-0.044487	0.670284
ApoB/ApoA1	0.130336	0.210537
Atrial rate	0.056227	0.602836
Ventricular rate	0.056227	0.602836
LAD	0.004660	0.965227
LVESD	0.111351	0.298865
LVEDD	0.022215	0.836295
LVESV	0.024295	0.823254

LVEDV	-0.043735	0.685774
LVEF	-0.177028	0.095083
**LVFS**	**-0.335167**	**0.001324**
LVMI	0.138107	0.210272
PWD	-0.100752	0.347499
E\`	-0.015914	0.883688
A\`	-0.026208	0.812934
S\`	-0.167885	0.254035
E\`/A\` ratio	0.004883	0.964841
E/A ratio	0.161625	0.130234
DT	-0.089404	0.404743
IVRT	-0.178607	0.094003
RVSP	0.007777	0.945061
RVEDD	**-0.210963**	**0.047203**
Right ventricular hypertrophy	0.011736	0.916658
IVSD	-0.141706	0.185288
Ascending aorta diameter	**-0.282342**	**0.007014**
IMC CCA left mean	0.022186	0.855344
IMC CCA right mean	-0.041853	0.732760
IMC CCA mean	-0.031564	0.795333
IMC ba left mean	**-0.296014**	**0.015009**
IMC ba right mean	-0.123406	0.319774
IMC ba mean	-0.222608	0.070199
PLA left mean	-0.165925	0.417891
PLA length left	0.221272	0.277338
PLA density left	0.007870	0.969563
PLA right mean	-0.115111	0.516815
PLA length right	0.009169	0.958956
PLA density right	-0.017732	0.920716
PLA mean	-0.095258	0.586216
PLA length	0.044033	0.790111
PLA density	0.030067	0.855819
ABI left	-0.104511	0.396347
ABI right	-0.122108	0.321206
ABI mean	-0.122194	0.320864

ABI – ankle-brachial index, BMI – body mass index, BP – blood pressure, DT – E-wave deceleration time, HR – heart rate, IMC CCA – intima-media complex of common carotid arteries, IMC ba — intima–media complex of brachial arteries, IVRT – isovolumic relaxation time, IVSD – interventricular septum at end-diastole, LAD – left atrium diameter, LVEDD – left ventricular end-diastolic diameter, LVEDV – left ventricular end-diastolic volume, LVEF – left ventricular ejection fraction, LVESD – left ventricular end-systolic diameter, LVESV – left ventricular end-systolic volume, LVFS – left ventricular fractional shortening, LVMI – left ventricular mass index, MAP – mean arterial pressure, PLA – plaque of common carotid artery bifurcation, PWD – posterior wall thickness at end-diastole , RBC – red blood cells, RVEDD – right ventricular end-diastolic diameter, RVSP – right ventricular systolic pressure, WBC – white blood cells, WHR – waist-to-hip ratio.

Table S2. The associations between qualitative variables and serum IL-6 concentration in early onset CAD patients.

Variable	IL-6
	**p-value**
History of hypertension	
Yes vs. No	0.828592
Past MI	
Yes vs. No	0.509520
Diastolic function	
normal vs. pseudonormal + impaired	0.697604
pseudonormal vs. normal + impaired	0.943436
impaired vs. normal + pseudonormal	0.790215
ABI	
<1.0 vs. ≥1.0	0,152972
IMC CCA	
≤ 0.9 vs. > 0.9	0,459809
IMC ba	
≤ 0.6 vs > 0.6	0,207399
Presence of PLA	
Yes vs. No	0,666665
PLA length	
≤ 6.0 vs. > 6.0	0,669043
PLA density	
≤ 70 vs. > 70.0	0,565681

IMC CCA – intima-media complex of common carotid arteries, IMC ba — intima–media complex of brachial arteries, MI – myocardial infarction, PLA – plaque of common carotid artery bifurcation.
